# Comparison of Color Stability and Surface Roughness of Interim Crowns Fabricated by Conventional, Milling and 3D Printing Methods

**DOI:** 10.1002/cre2.70119

**Published:** 2025-04-22

**Authors:** Zahra Hashemzade, Mohammad Alihemmati, Seyed Mohammad Reza Hakimaneh, Sayed Shojaedin Shayegh, Mohammad Amin Bafandeh, Zahra Mohammadi

**Affiliations:** ^1^ Department of Prosthodontics, Faculty of Dentistry Shahed University Tehran Iran

**Keywords:** color stability, provisional crowns, surface roughness

## Abstract

**Objectives:**

Manufacturing temporary restorations is part of the treatment process in fixed prostheses, which is accomplished by different methods. The aim of this study is to compare the color stability and surface roughness of provisional crowns made by 3D printing, conventional and milling methods.

**Materials and Methods:**

In this semi‐experimental study, 60 provisional crowns were created by conventional, milling, and 3D printing methods (20 samples for each method). Half of the samples in each group were treated with BisCover surface sealant after construction. The surface roughness was checked using a laser profilometer. To determine the color stability, an evaluation was done using a spectrophotometer on the first day and the second and fourth weeks after exposure to the tea solution, and ∆*E* was calculated using L*a*b* values. Statistical analysis was performed at a significance level of 0.05.

**Results:**

The surface roughness in the conventional group was significantly higher than in the milling group (*p* < 0.05). The surface‐treated samples had less surface roughness and more color stability than other samples (*p* < 0.05). ∆E in the 3D printing group was higher than other groups in all time intervals (*p* < 0.05).

**Conclusion:**

The milling method can be considered the best method of making provisional crowns in terms of color stability and surface smoothness. Also, the use of sealing materials can have a significant effect on improving color stability and surface smoothness in provisional crowns made by any method.

## 1 Introduction

An interim or provisional restoration is a dental prosthesis that can be fixed or removable, designed to improve esthetics, stabilization, and/or function for a limited period of time, and then it has to be replaced by a definitive restoration (Ferro et al. 2017). In cases of full mouth reconstruction where multiple teeth need to be restored, provisional restorations are especially important, as they protect the pulp tissue from thermal, mechanical, physical, and bacterial triggers (Abdullah et al. 2018; Alt et al. 2011). A well‐constructed provisional restoration is also esthetically important and keeps the soft tissue intact until the definitive restoration is delivered (Digholkar et al. 2016; Giti et al. 2019). There are different techniques for making provisional restorations, which are different in terms of accessibility and costs.

The conventional method involves mixing powder and liquid on the external surface, which can lead to pores and adversely affect the mechanical and surface properties. Some of the common disadvantages of the conventional method include polymerization shrinkage, thermal damage to pulp cells, porous surface, lack of marginal adaptation, water absorption, and color instability (Alt et al. 2011). Also, this technique relies a lot on the skill of the technician and is time‐consuming, especially for making Multi‐Unit Fixed Dental Prostheses (W.‐S. Lee, D.‐H. Lee, and K.‐B. Lee 2017; Simoneti et al. 2022).

Computer‐aided design/computer‐aided manufacturing (CAD–CAM) is increasingly being used to create dental restorations. CAM technology includes subtractive and additive manufacturing methods. Additive method is also called 3D printing method. In the subtractive manufacturing method, temporary restorations are milled from a prefabricated polymethyl methacrylate (PMMA) block with a high degree of conversion, precision, strength, marginal adaptation, and high color stability (Rayyan et al. 2015). However, some defects, such as positive and negative errors in the diameter of the milling bur cause inaccuracy, and in addition, material wastage, and the inability to create complex milled shapes in CAD–CAM technique are among the disadvantages (Alharbi et al. 2016; Lee, Jun, et al. 2017; Park et al. 2016; Scotti et al. 2020). In the additive manufacturing system, the product is made by continuous accumulation of powder and liquid type of materials (Garg et al. 2021; Revilla‐León et al. 2019). In this technique, fewer materials are used, and it is affordable. In addition, this technology can produce more complex structures compared to the milling technique (Digholkar et al. 2016; Alharbi et al. 2016; Park et al. 2016).

Accumulation of biofilm on dental materials causes gingivitis and secondary caries. Furthermore, surface roughness causes the accumulation of microbial plaque and increases the possibility of caries (Krzyściak et al. 2014). Compared to definitive restorations, provisional restorations have higher surface roughness and less marginal adaptation, which causes more biofilm adhesion on their surfaces (Buergers et al. 2007; Köroğlu et al. 2016). Discoloration of temporary restorations during long‐term treatment may lead to patient dissatisfaction and additional costs for replacement (Sham et al. 2004). Therefore, color stability is an important factor in the selection of temporary restoration materials, especially in the cosmetic area. Several factors, such as incomplete polymerization, water absorption, chemical reaction, surface roughness, diet, and oral hygiene may affect the degree of discoloration (Rutkunas et al. 2010).

Proper polishing provides a smooth surface for the restoration; however, the polishing process may cause cracks or defects on the restoration surface. Using a surface sealant can improve the characteristics of the restoration surface, however, there are reports of debonding or wear of the surface sealant in studies (Mazurek‐Popczyk et al. 2022). Many studies compared the mechanical properties of different temporary materials according to their manufacturing methods (Abdullah et al. 2018; Scotti et al. 2020; Reymus et al. 2020). However, limited information is available on the biological behavior of provisional restorations fabricated through these methods. Hence, the present study aims to compare the color stability and surface roughness of provisional crowns made by 3D printing, conventional and milling methods. The null hypothesis of this article is that there is no difference in surface roughness and color stability of temporary veneer made by three common methods: milling, 3D printing, and conventional.

## 2 Methods

**Figure 1:**
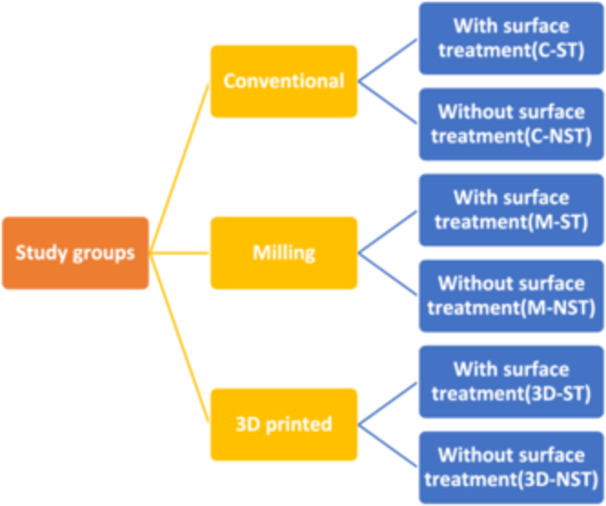
Study groups flowchart.

To determine the appropriate sample size for the study, a G*Power analysis was performed. Based on the results, 20 samples were included in each group. These samples were further divided into two subgroups to assess the impact of surface treatment on both surface roughness and color. The study design is illustrated in Figure 1.

Initially, the maxillary molar resin tooth was prepared based on all‐ceramic grinding steps using a Tapered torpedo diamond bur (D&Z, Germany) with 1.5 mm occlusal grinding, 1 mm axial grinding, and 1 mm shoulder finishing line. Then, the trimmed resin tooth was scanned using a 3D laboratory scanner (shining 3D DS‐EX pro, China) and the prepared resin tooth was made by a 3D printer. Also, due to the heat of polymerization during the manufacturing of conventional crowns and the possibility of damage to the printed model, a milled zirconia model (Xtcera, China) was also considered.

A total of 60 provisional crowns were produced using three techniques including 3D printing, milling, and conventional methods. The printed PMMA resin model was scanned with a scanner (shining 3D DS‐EX pro, China).

To create conventional provisional crowns, the PVS index was developed using the milled zirconia model. Subsequently, the Tempron powder and liquid were blended and placed in the index before being applied to the zirconia model. After manufacturing the 20 conventional crowns, finishing and polishing procedures were performed on all samples. To fabricate the milled provisional crowns, the STL file data of the printed model was used, and the design of the temporary restorations was carried out using the Exocad software. CAD‐CAM provisional crowns were milled from polymethyl methacrylate blocks (PMMA A1, China OD 98x16mm LOT NO20180610) using a 5‐axis milling machine (Xtcera Xmill 500 China). In the 3D printing group, temporary crowns were made with special methacrylate printing material (FREEPRINT Temp ‐at DETAX, Germany) by a 3D printer (Asiga MAX 3D Printer, 3D Drucker Nach Marke Sydney, Australia) using the DLP technique (Table 1). All the samples in three groups were polished using three molts of aluminum oxide after fabrication. After polishing, half of the samples of each group were subjected to surface treatment using the surface sealant BisCover LV, Bisco Inc. (Rayyan et al. 2015). After applying a thin layer of BisCover LV, the samples were light‐cured for 30 s at close range (0–2 mm).

**Table 1:** Materials used in this study.

Group	Product	Manufacturer	Shade	Country	Type
Conventional‐NST	Tempron	GC Corporation	A1	Japan	Autopolymerizing resin
Conventional‐ST					
Milled‐NST	OD98mm PMMA Block	YUCERA	A1	China	CAD‐CAM polymethyl methacrylate resin
Milled‐ST					
3D printed‐NST	FREEPRINT® Temp	DETAX	A1	Germany	Methacrylate‐based resin
3D printed‐ST					
—	BisCover LV	Bisco	—	USA	Dipentaerythritol pentaacrylate, ethanol, camphorquinone

The surface roughness was studied using a surface laser profilometer in the occlusal, middle, and gingival third of the buccal surface of each restoration. To evaluate the color stability, after determining the initial color of the restorations in the mid‐buccal area with a fixed background by spectrophotometer, the samples were placed in a tea solution in a 37‐degree incubator for 2 and 4 weeks, which is equivalent to 2.5 years of the clinical exposure of the restoration. The solution was changed every 2 days. At each measurement time, they were washed with distilled water for 5 min and measured by an X‐Rite Gretag Macbeth COLOR‐EYE 7000 A spectrophotometer (Guler et al. 2005). In this study, to measure the reflectance spectrum and color coordinates of the samples, the spectral range of the spectrophotometer was set from 400 to 750 nm with 10 nm intervals. Also, SCI mode and aperture size of 1 cm were selected. The data subjected to statistical analysis at a significance level of 0.05.

The color difference value (∆E) in the CIELAB color space is calculated using the following formula:

$$∆E=\sqrt{{\left({L^{*}}_{2}-{L^{*}}_{1}\right)}^{2}+{\left({a^{*}}_{2}-{a^{*}}_{1}\right)}^{2}+{\left({b^{*}}_{2}-{b^{*}}_{1}\right)}^{2}}$$

where *L* ^*^ represents lightness, with higher values indicating greater brightness. *a* ^*^ represents the green‐red spectrum, where negative values indicate green and positive values indicate red. *b* ^*^ represents the blue‐yellow spectrum, where negative values indicate blue and positive values indicate yellow.

## 3 Results

60 provisional crowns were made using three techniques, and in each group, half of the samples were subjected to surface treatment with sealant. The results of surface roughness comparison between groups using the ANOVA test showed a significant difference between manufacturing techniques (*p* < 0.001).

**Figure 2:**
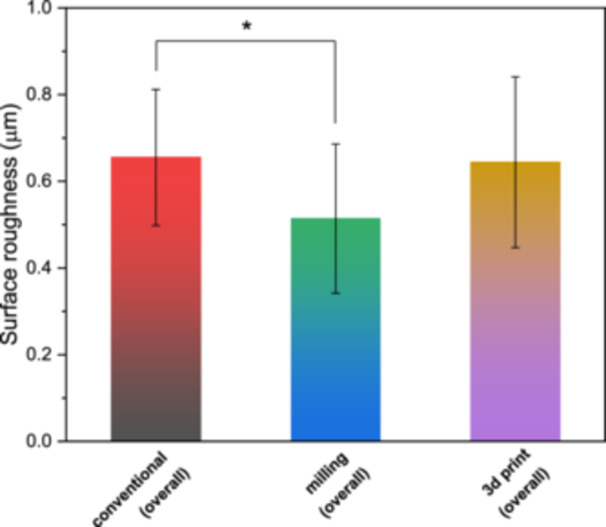
The overall average surface roughness of different groups with and without surface treatment. The star indicates a statistically significant difference.

According to Table 2, the highest surface roughness was reported for the conventional samples with an average Ra of 0.655 ± 0.157. Following that, the 3D printed and milled samples showed the highest surface roughness, respectively. The results of the ANOVA statistical test showed a significant difference between the groups in terms of surface roughness (*p* = 0.025). As shown in Figure 2, the results of the Tukey post hoc test revealed that the average surface roughness of the temporary crowns made by the conventional method was significantly higher than the milling group (*p* = 0.037). Among the crowns made by the conventional and milling methods, the subgroup with surface treatment had significantly lower surface roughness (*p* < 0.001). However, among the samples made via 3D printing, there was no significant correlation between surface roughness and surface treatment (*p* = 0.354).

**Table 2:** Surface roughness in the studied groups.

	Ra (μm) based on surface treatment			
	Conventionally polished groups		BisCover LV‐coated groups	
Groups	**Mean ± SD**	**Min‐Max (Median)**	**Mean ± SD**	**Min‐Max (Median)**
Conventional	0.796 ± 0.075^Aa^	0.65–0.90 (0.782)	0.515 ± 0.049^ABb^	0.41–0.59 (0.528)
Milled	0.650 ± 0.081^Ba^	0.51–0.78 (0.673)	0.380 ± 0.122^Ab^	0.24–0.63 (0.337)
3D printed	0.686 ± 0.250^ABa^	0.41–1.20 (0.640)	0.602 ± 0.123^Ba^	0.45–0.78 (0.595)

*Note:* Mean difference significant at *p* < 0.05; Means with identical letters do not differ statistically. Uppercase and lowercase letters are used for columns and rows, respectively

**Figure 3:**
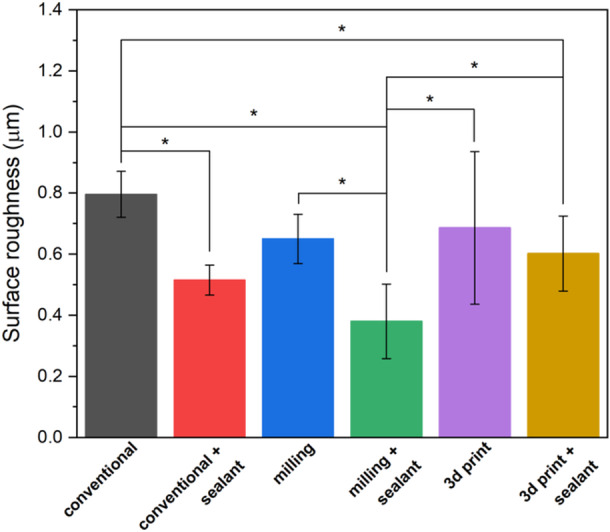
Surface roughness of different groups with different surface treatment. The stars indicate statistically significant differences.

The results of Tukey's post hoc test showed that among the crowns without surface treatment, the surface roughness in the temporary crowns made by the conventional method (0.796 ± 0.075) was significantly higher than the milling group (0.650 ± 0.081) (*p* < 0.05) (Figure 2). Also, among the BisCover LV‐coated groups, the surface roughness of the temporary crowns made by the milling method (0.380 ± 0.122) was significantly lower than the 3D printed group (0.602 ± 0.123) (*p* < 0.05) (Figure 3). BisCover LV‐coated groups of crowns fabricated by conventional and milling methods had significantly less surface roughness than their conventionally polished counterparts (*p* < 0.05) (Table 2 and Figure 3).

**Figure 4:**
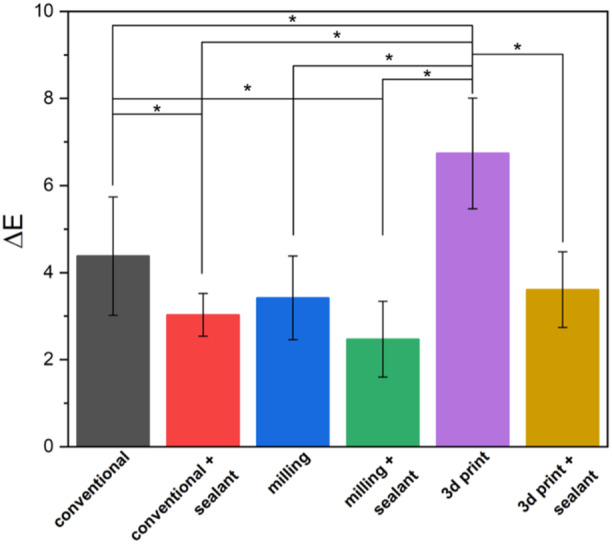
Color change for the first two weeks period (weeks 1 and 2). The stars indicate statistically significant differences.

**Figure 5:**
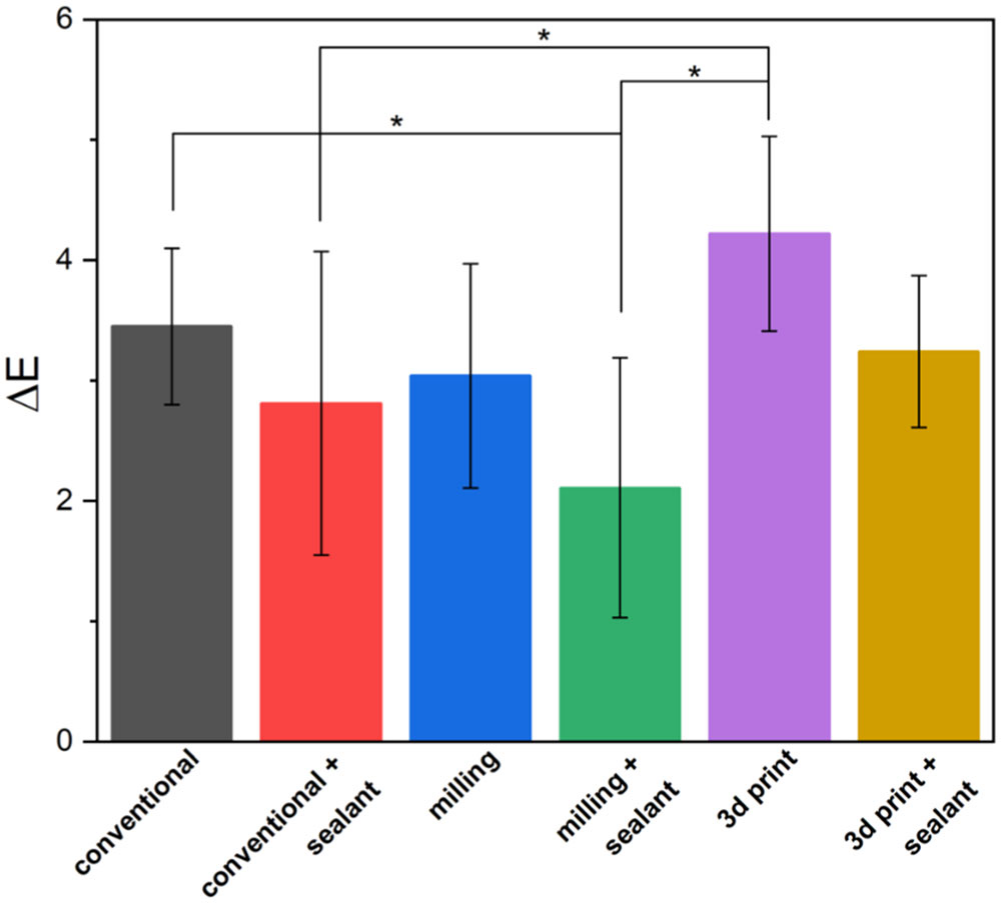
Color change for the second two‐week period (weeks 3 and 4). The stars indicate statistically significant differences.

**Figure 6:**
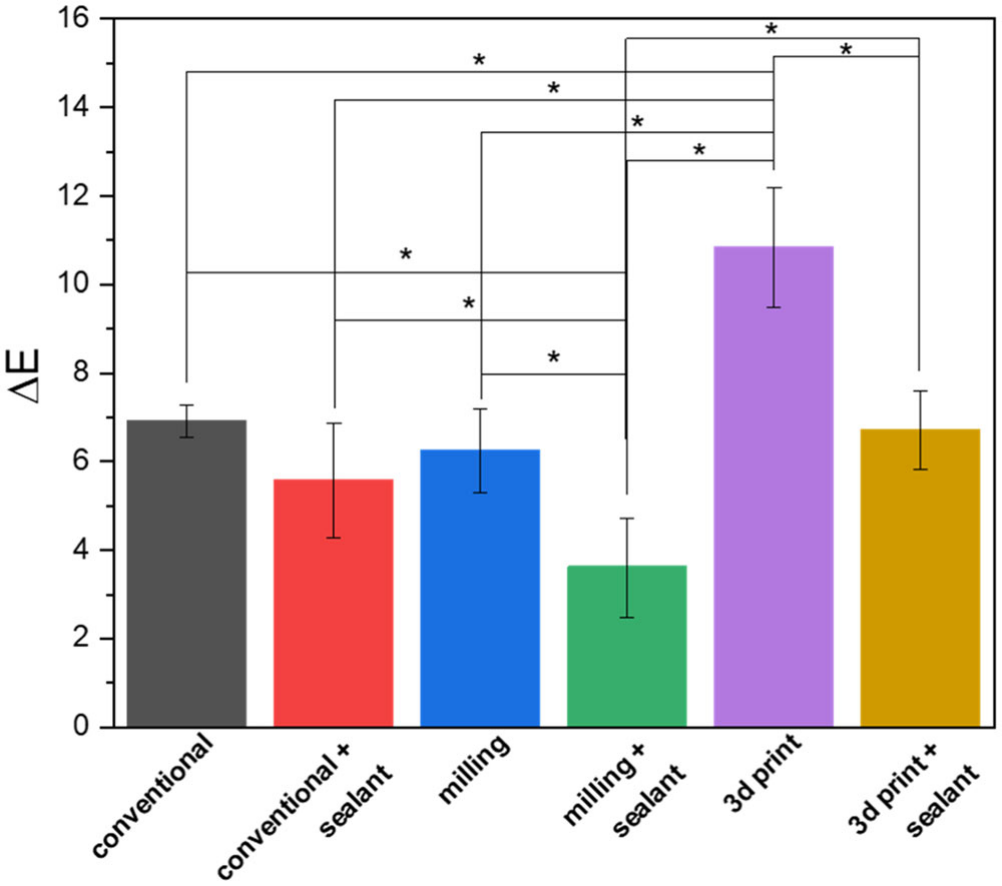
Color change for the entire 4‐week period. The stars indicate statistically significant differences.

Color changes for different groups and different times are presented in Table 3. Color changes up to the second week in 3D printed crowns (6.74 ± 1.27) were significantly more than other groups (*p* < 0.05). Color changes in BisCover LV‐coated crowns, made by conventional (3.03 ± 0.49) and 3D printing methods (3.61 ± 0.87) were significantly less than polished counterparts (4.38 ± 1.36 and 6.74 ± 1.27, respectively) (*p* < 0.05) (Figure 4). The color changes for the second 2 weeks (weeks 3 and 4) in the BisCover LV‐coated crowns made by the milling method (2.11 ± 1.08) were significantly less than the conventional (3.45 ± 0.65) and 3D printed groups (4.22 ± 0.81) (*p* < 0.05) (Figure 5). For the entire 4‐week period, the color changes in the 3D printed crowns (10.84 ± 1.35) were significantly higher than in other groups (*p* < 0.05). Color changes up to the fourth week in BisCover LV‐coated crowns made by milling (3.61 ± 1.12) and 3D printing methods (6.71 ± 0.88) were significantly less than polished counterparts (6.24 ± 0.95 and 10.84 ± 1.35, respectively) (*p* < 0.05) (Figure 6)

**Table 3:** Color changes in the studied groups.

		Color difference values (∆E)				
		Conventionally polished groups		BisCover LV‐coated groups		
Time period	**Method**	**Mean ± SD**	**Min‐Max**	**Mean ± SD**	**Min‐Max**	***p*‐value**
First two weeks (weeks 1 and 2)	Conventional	4.38 ± 1.36	3.10–6.91	3.03 ± 0.049	2.42–3.70	0.013
	Milled	3.42 ± 0.96	2.13–4.75	2.47 ± 0.87	1.00–3.91	0.033
	3D printed	6.74 ± 1.27	5.47–8.76	3.61 ± 0.87	2.16–4.92	< 0.001
Second two weeks (weeks 3 and 4)	Conventional	3.45 ± 0.65	2.43–4.23	2.81 ± 1.26	0.77–4.81	0.010
	Milled	3.04 ± 0.93	1.81–4.69	2.11 ± 1.08	0.59–3.45	< 0.001
	3D printed	4.22 ± 0.81	3.35–5.49	3.24 ± 0.63	2.26–4.36	< 0.001
Entire four‐week period (weeks 1–4)	Conventional	6.91 ± 0.36	6.27–7.38	5.57 ± 1.30	3.31–7.10	0.010
	Milled	6.24 ± 0.95	4.66–7.67	3.61 ± 1.12	1.57–5.13	< 0.001
	3D printed	10.84 ± 1.35	9.11–12.40	6.71 ± 0.88	5.00–7.68	< 0.001

The evaluation of color stability showed that the color changes for the first and second 2 weeks and the entire 4 weeks, has a significant relationship with the manufacturing method and surface treatment. In all time intervals, the color change in samples made with 3D printing was significantly higher than the other groups. The results of the independent test showed that the color change in the samples that were treated with sealant was significantly less than the samples without sealant. In Figures 4, 5, 6, color changes are shown separately for each group.

## 4 Discussion

The present study was conducted with the aim of comparing the color stability and surface roughness of temporary crowns made by 3D printing, conventional, and milling methods. In general, 60 samples were made (20 samples of each method) and half of the samples of each group were subjected to surface treatment using BisCover LV surface sealant.

In the literature, two techniques are mainly used to investigate the color change in dental restorations: Accelerated aging and immersion in different solutions (Almejrad et al. 2022). In this study, the immersion method in the tea solution was used to examine the color stability.

Goldstein et al. reported that a Δ*E* of 3.7 or greater exceeds the clinical tolerance limit (Goldstein and Schmitt 1993). A Δ*E* of 3.7 or less is visually inconspicuous and also clinically acceptable (Taşın et al. 2022). If the samples are considered separately into six groups, the milling + surface sealant group showed an average color change of 3.61 ± 1.12, which is at the threshold of clinical tolerance. However, the average color changes in other groups were higher than 5.5. Similar findings were reported in Song et al.'s study, which aimed to investigate the color stability of disks prepared from five different materials of temporary crowns by conventional, 3D printing, and milling methods. In their study, PMMA milling discs showed similar results for 4 weeks, but the color difference increased sharply after 8 weeks and showed less stability in long‐term testing. The most color change was caused by the tea solution in the 3D printing group (Song et al. 2020). These findings were consistent with the present study. In our study, the samples were examined for 4 weeks. Which is equivalent to 2.5 years of clinical exposure. 3D printed samples showed the most color change; So, comparing the results of the second week and the fourth week with the first day, the lowest color stability with a relatively large difference was related to the 3D printed samples. This result may be due to the uncured layer remaining even after post‐curing due to the characteristics of 3D printing. The highest level of color stability in the present study was related to the samples made by the milling method with surface treatment.

Elagra et al. reported that CAD/CAM PMMA materials provide better color stability compared to various types of conventionally fabricated temporary restorations (Elagra et al. 2017). In addition, Atria et al. (2020) reported that the amount of color change based on the CIEDE2000 criteria was the lowest in the milling group and the highest in the 3D printing group (Atria et al. 2020). These findings are completely consistent with the results of the present study. Al‐Akhali et al. (2023) similarly reported the least color change in milled samples but reported the highest color change in conventional light‐cured composite samples. Incongruously, in the present study, the 3D printed samples showed the most color change during 2 and 4 weeks. The reason for this difference could be the shorter duration of the evaluation by Al‐Akhali et al. (1 week) or the difference in the exposure environment (distilled water solution). It is obvious that the color change will be more intense after exposure to colored solutions and for a long time. Also, a recent study by Rizzante et al. in 2023 showed that provisional crown samples made through 3D printing provide similar color stability to samples made by conventional methods (Almejrad et al. 2022). These findings were not consistent with our results. However, this study was also different from the present study in terms of the type of materials used in the conventional method and the time period of color change evaluation, which can have a significant impact on the results. However, as mentioned above, studies that had a similar design to the current research have shown the samples made by 3D printing as the weakest samples in terms of color stability. Based on past studies, poor color stability of 3D printing resins has been attributed to higher polymer hydrophilicity/polarity, lack of filler particles, increased surface roughness, presence of residual monomers, high solubility, and dependence on post‐fabrication materials and protocols (Song et al. 2020; Atria et al. 2020).

In the present study, the samples that were subjected to surface treatment with sealant showed better color stability than the merely polished samples. Similar findings were reported by Yao et al. (2021), where the least color change was reported in the milling group with optiglaze treatment. The color change in the surface‐treated samples was significantly less than the samples without surface treatment. The difference between the optiglaze and skinglaze surface sealant groups was not significant. They showed that the use of sealants can significantly reduce the discoloration of provisional dentures (Yao et al. 2021). The use of a surface sealant may strengthen the resistance to discoloration in the samples by reducing surface irregularities and defects (Şahin et al. 2015).

Tasın et al. (2022) conducted a study on surface roughness and color stability of disks made by conventional, milling, and 3D printing methods. Similarly to the present study, half of the discs of each group were subjected to surface treatment after polishing with BisCover LV sealant. Color change was measured one, 7 and 30 days after exposure to colored solutions and distilled water. All the groups that received surface sealant had more color stability than the groups without surface treatment. The least color change was reported in the milling group with sealant surface treatment, and the most intense color change was shown in the conventionally made PMMA group (Taşın et al. 2022). In the present study, the least color change was reported in the groups receiving surface sealant, but in the comparison between the manufacturing methods, the most color change was observed in the 3D printing group. The inconsistency with the above study can be related to the material used to make the samples, as it was said in the study of Tasin et al. resin composite material was used for 3D printing, but in the present study, polymethyl methacrylate was used for this purpose.

In the present study, all the values of surface roughness were higher than the determined threshold (0.2 μm), which causes biofilm accumulation, but these values were less than the clinically unacceptable limit (10 μm) (Köroğlu et al. 2016). In the present study, the samples that were made by milling method and were subjected to surface sealant treatment showed the least surface roughness. The highest surface roughness belonged to conventional samples. In 2020, Atria et al. showed that the surface roughness of provisional crown samples made by milling method was lower than conventional and 3D printed samples (Atria et al. 2020). These findings were consistent with the results of the present study. Surface roughness is one of the most important factors that affect biofilm accumulation on dental materials (Dantas et al. 2016). The study of Gitti et al. showed that the samples made by the conventional method showed a significantly higher surface roughness than the other groups, and the milling samples had the lowest surface roughness, which was not significantly different compared to the 3D printed group (Giti et al. 2021). In the current study, the surface roughness of the 3D printed restorations was significantly higher than the milling group, and this difference in the results of the two studies could be due to the difference in the polymethyl methacrylate block used for the milling samples or the geometrical difference of the dental crowns and the fabricated rods made of temporary crown materials. In the study of Gitti et al. a contact profilometer was used to evaluate the surface roughness, but in the present study, a laser profilometer (noncontact) was used.

In the study of Aldahian et al. (2021), the surface roughness of 3D printed provisional crowns was reported to be the highest surface roughness compared to those made by conventional and milling methods (Aldahian et al. 2021), which was contrary to the results of the present study. This discrepancy may be due to differences in the raw material or differences in the 3D printer or other laboratory aspects of the preparation of restorations. The results of another study in 2021 by Al‐Qahtani et al. were similar to the results of Aldahian et al.'s study (Al‐Qahtani et al. 2021).

Meshni et al. (2018). reported higher surface roughness in conventionally cured PMMA resins than in modified methyl methacrylate resins and CAD‐CAM PMMA blocks. They observed the lowest surface roughness in CAD‐CAM PMMA blocks. These findings were consistent with the results of the present study, which show that the conventional manufacturing method creates more surface roughness, and the use of milling methods by CAD‐CAM systems can create less surface roughness. Simoneti et al. (2022) and Tasin et al. also showed more surface roughness in the conventional manufacturing method. Presumably, the high surface roughness of the conventional group is due to air bubbles created by manual mixing of the liquid and powder during the manufacturing process (Alt et al. 2011; Köroğlu et al. 2016). Also, in the study of Tasin et al. the groups that received the BisCover LV surface treatment had the lowest amount of surface roughness, and among the fabrication methods, the lowest surface roughness was observed in the 3D printing group. In this study, the superiority of the 3D printed group over milling was attributed to the process of block milling and the possibility of creating an uneven surface during milling (Taşın et al. 2022). As mentioned earlier, the difference in the results of the present study and the study of Taşin et al. can be related to the difference in the material used and the method and steps of the study design.

In the present study, the findings indicated that in terms of surface roughness, the surface‐treated samples had much less surface roughness than other samples. Sealant agents are recommended to improve the optimal properties and especially the surface smoothness of restorations by filling micro‐cracks and micro‐defects that are created after the finishing/polishing process. However, sealant materials may have problems such as low wear resistance, poor adhesion to the underlying material, and poor surface quality due to uneven spreading (Bertrand et al. 2000; Sarac et al. 2006).

The study has several limitations. First, the in vitro design may not fully replicate the complexities of the in vivo oral environment. Additionally, by focusing on a single factor (Taşın et al. 2022; Song et al. 2020), the impact of an individual variable on surface roughness and color stability was precisely assessed, but potential interactions between multiple variables in clinical settings were not addressed. The exclusive use of tea as the testing beverage, chosen for its prevalence in Iran, may limit the generalizability of the findings to populations with different dietary habits. Furthermore, the construction of temporary crowns involves numerous parameters (e.g., technician precision, 3D printer type, object size, layer thickness, construction angles, support methods, and milling machine precision) that can affect the final outcome, thereby necessitating larger sample sizes to examine each factor comprehensively. Finally, the high costs associated with laboratory procedures further constrained the research.

## 5 Conclusion

1. All groups had surface roughness above the plaque accumulation threshold (0.20 μm), but their surface roughness was below the clinically unacceptable value of 10 μm.
2. The use of BisCover LV surface sealant significantly reduces the surface roughness and improves the color stability of the samples.
3. The milling method can be considered as the best method of manufacturing temporary crowns in terms of color stability and surface smoothness.

## Author Contributions

Zahra Hashemzade conducted the research, performed the experimental work, and wrote the thesis and the initial draft of the manuscript. Mohammad Alihemmati supervised the project, providing guidance throughout the research process. Seyed Mohammad Reza Hakimaneh, Sayed Shojaedin Shayegh, and Mohammad Amin Bafandeh contributed to advisory roles, offering valuable insights and expertise. Zahra Mohammadi reviewed and edited the final manuscript, ensuring its readiness for submission. All authors reviewed and approved the final version of the manuscript.

## Conflicts of Interest

The authors declare no conflicts of interest.

## Data Availability

The data that support the findings of this study are available from the corresponding author upon reasonable request.
